# Spectrum of Geriatric Predominant Epithelial Malignancies in Western India: A Prospective Histopathological Analysis

**DOI:** 10.7759/cureus.106302

**Published:** 2026-04-01

**Authors:** Amit Agravat, Vikas Chauhan, Raghunath Baria, Hetvi Unadkat, Jashswee Bora, Gauravi Dhruva, Tarang Patel

**Affiliations:** 1 Pathology, Pandit Dindayal Upadhyay Medical College, Rajkot, Rajkot, IND; 2 Pathology, All India Institute of Medical Sciences (AIIMS) Rajkot, Rajkot, IND

**Keywords:** adenocarcinoma, cancer, geriatric patient, histopathology examination, squamous cell carcinoma

## Abstract

Introduction

Geriatric cancer is mostly diagnosed in elderly people in the age group of more than 60. Aging is the strongest predictor of cancer risk. Beyond the late 80s or around 90 years of age, the incidence of newly diagnosed cancers declines, while morbidity and mortality due to cardiovascular and other age-related conditions increase. Oral cancers represent one of the most common malignancies in this region and are also a major contributor to the global cancer burden.

Methods

In this study, biopsy samples were collected from patients aged 60 years and above who were diagnosed mainly with epithelial malignancies. These samples were examined at the histopathology laboratory of a tertiary care hospital in Rajkot, Western India. All specimens were routinely processed for microscopic evaluation, and immunohistochemistry (IHC) was performed in selected cases whenever additional confirmation was needed.

Results

In the present study, a total of 111 cases were studied. The maximum number of geriatric cancer cases was observed in the 60-65-year age group, comprising 55 cases (49.5%). The squamous cell carcinoma was the predominant malignancy, accounting for 65 (58.6%) of the total cases. This was followed by adenocarcinoma, which constituted 14 (12.6%) of the cases. Immunohistochemical analysis was utilized in selected cases based on diagnostic necessity.

Conclusion

The findings of this study contribute to a better understanding of the histopathological spectrum of geriatric cancers. These results may aid in improving diagnostic accuracy and support the development of more effective management strategies for cancer in the elderly population.

## Introduction

Cancer in older adults, or geriatric cancer, primarily affects individuals aged 60 years and above and accounts for a growing share of global cancer cases and deaths [1]. Aging is recognized as the primary risk factor for the development of cancer, with prevalence increasing steadily as adults grow older. Epidemiological studies indicate that cancer incidence peaks around 85-90 years of age, after which it tends to decline, while the occurrence of cardiovascular and other age-related diseases rises [2,3].

Aging is not solely defined by the number of years lived. Chronological age may differ from biological age, which reflects physiological and cellular markers of aging. Individuals with a younger biological age often show better health and lower susceptibility to cancer [4]. Some researchers have proposed that, while aging itself is unavoidable, modifiable biological processes - such as metabolic health, lifestyle habits, and immune function - can influence cancer risk [5]. Understanding these aspects is particularly relevant in elderly patients, as it helps contextualize the histopathological patterns observed in our study and highlights opportunities for targeted prevention and tailored management in this population.

Lifestyle factors that slow the aging process - such as maintaining a healthy body weight, consuming a predominantly plant-based diet, engaging in regular physical activity, and avoiding exposure to environmental carcinogens - appear to reduce the likelihood of developing cancer [6]. Although these associations do not establish causation, they suggest that the biological processes underlying aging and cancer may overlap or that advanced biological aging can accelerate the development of clinically detectable tumours that manifest as age-related diseases [5].

The aging process is marked by a progressive decline in the function of multiple organ systems, which varies between individuals and even within the same individual over time [7]. Cancer predominantly affects older adults, with more than 80% of cases occurring in people over 50 years of age [8]. The cellular and molecular mechanisms that increase cancer susceptibility - such as DNA damage accumulation, genomic instability, and altered tissue repair - also contribute to other age-related chronic conditions [7,9]. One of the key contributors is immunosenescence, the gradual decline in immune system function that occurs with age. This weakened immune surveillance reduces the body's ability to identify and eliminate abnormal cells, increasing cancer susceptibility. Cancer is commonly viewed as an age-associated disease due to the accumulation of genetic mutations over time, reduced DNA repair capacity, increased sensitivity to carcinogens, and enhanced carcinogenic processes in aged tissues [8,10].

Older adults with cancer often exhibit more aggressive tumor behavior and face additional challenges, including higher treatment toxicity, multiple comorbidities, reduced physiological reserve, and limited access to comprehensive care [1,11]. These factors collectively contribute to increased mortality among older patients compared with younger populations [1,11]. Approximately 60% of new cancer cases and 70% of cancer-related deaths occur in adults aged 65 years or older [12,13]. Standard therapeutic protocols designed for younger patients may not be directly applicable, necessitating individualized approaches that balance efficacy with tolerability [14]. Furthermore, age-related pharmacokinetic and pharmacodynamic changes can alter drug metabolism, increasing the risk of toxicity, while social and cognitive factors may limit treatment adherence [15].

In India, the burden of geriatric cancers is rising alongside demographic shifts. Population-based registries show increasing incidence of cancers, such as breast, lung, gastrointestinal, and genitourinary malignancies in older adults [4,14]. However, region-specific data focusing specifically on elderly patients remain scarce, particularly in Western India. Understanding the clinicopathological characteristics of cancers in this population is essential to optimize diagnostic approaches, guide management, and inform healthcare planning [15].

## Materials and methods

Biopsy samples were taken from cancer patients aged 60 years and above at a tertiary care hospital in Rajkot, western India. These samples were examined using routine histopathological techniques, and in some cases, immunohistochemistry (IHC) was used to help confirm the diagnosis.

Study population

The study included all patients aged 60 years and above who were newly diagnosed with cancer during the study period. A total of 111 cases were analyzed after applying appropriate inclusion and exclusion criteria (Table 1).

**Table 1:** Inclusion and exclusion criteria

Category	Criteria
Inclusion Criteria	Patients aged ≥60 years diagnosed with malignant epithelial lesions
	Biopsy or surgical specimens adequate for histopathological evaluation
Exclusion Criteria	Specimens that were unsatisfactory or insufficient for definitive histopathological diagnosis
	Non-epithelial malignancy

Data collection

All specimens were collected and submitted to the histopathology laboratory in 10% formalin. Routine processing and hematoxylin and eosin (H&E) staining were performed. Histopathological examination was conducted using a light microscope by experienced pathologists to identify the type and characteristics of the malignancy.

IHC was performed selectively in cases where additional diagnostic confirmation was required. Data regarding age, sex, and site of lesion were recorded for each patient. Comparative analysis with previous regional and national studies was performed to contextualize the findings.

Ethical considerations

The study was approved by the Institutional Ethics Committee. Before participation, all study subjects provided written informed consent.

## Results

A total of 111 cases were diagnosed as geriatric cancers and included in the study during the period from October 2023 to April 2025.

In the present study, the highest number of geriatric cancer cases was observed in the 60-65-year age group, comprising 55 cases (49.5%) (Table 2). 

**Table 2:** Age-wise distribution of the cases

Age group (in years)	Male	Female	Total	Percentage (%)
60-65	30	25	55	49.5
66-70	18	04	22	19.8
71-75	14	06	20	18.1
76-80	02	05	07	6.3
80-85	03	04	07	6.3
Total	67	44	111	100

Among all cases, the most common site was the oral cavity, with 53 cases (47.75%), followed by the gastrointestinal tract with 20 cases (18.02%) and the breast with 13 cases (11.71%) (Table 3).

**Table 3:** Distribution of geriatric cancers according to site

Sr. No.	Site	Number of cases	Percentage (%)
1	Oral cavity	53	47.75
2	Gastrointestinal tract	20	18.02
3	Breast	13	11.71
4	Lung	5	4.50
5	Nose and paranasal sinus	4	3.60
6	Thyroid	3	2.70
7	Soft tissue	2	1.80
8	Skin	2	1.80
9	Eyelid	2	1.80
10	Neck region	3	2.70
12	Uterus	1	0.90
13	Prostate	1	0.90
15	Temporo-parietal region	1	0.90
16	Upper lip	1	0.90
Total		111	100

In the present study, squamous cell carcinoma was the most prevalent malignant condition, accounting for 65 cases (58.6%). The majority of squamous cell carcinoma cases were located in the oral cavity, with 53 cases (81.5%). The maximum number of cases was seen in the tongue (26 cases), followed by the buccal mucosa (21 cases) and the hard palate (6 cases).

**Figure 1:**
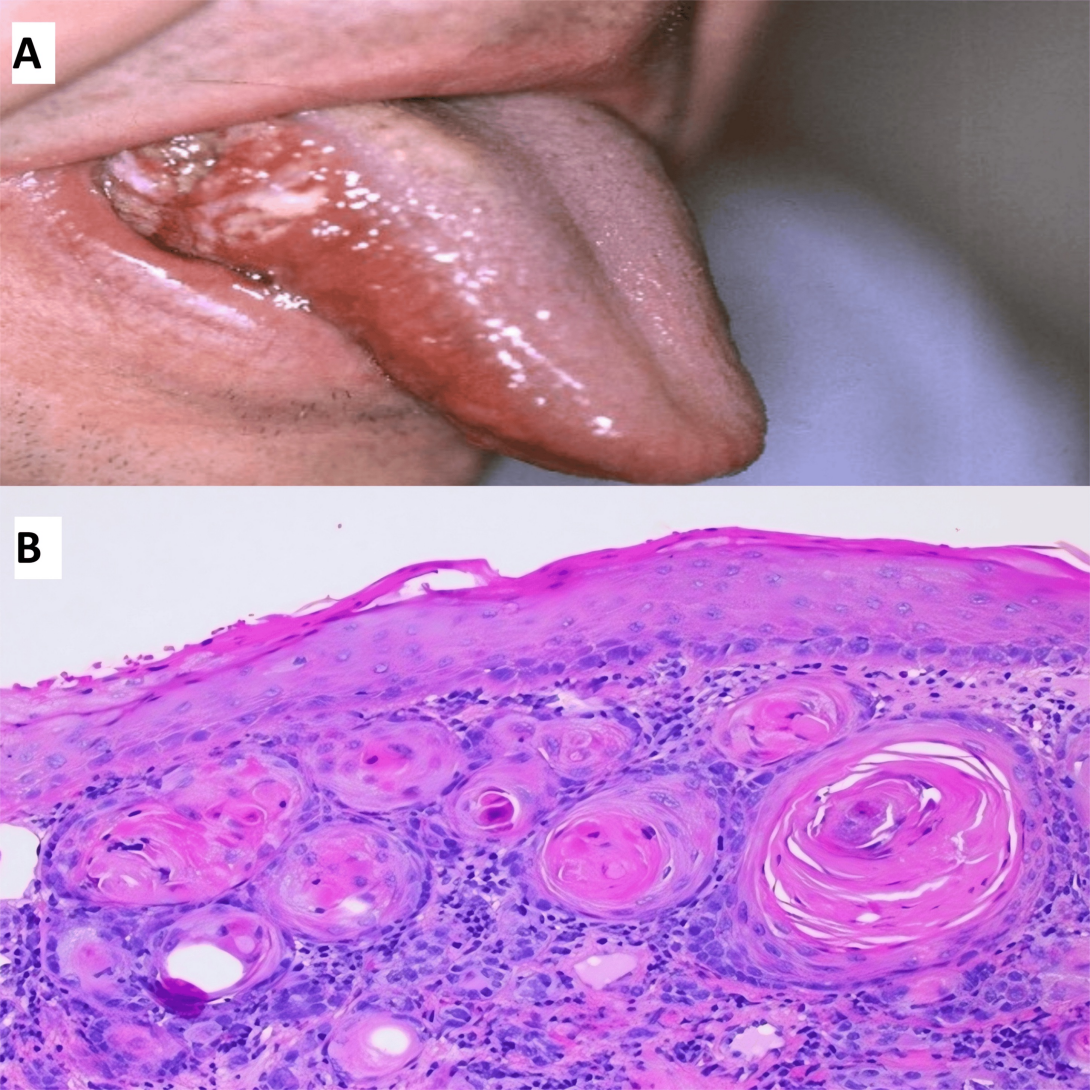
Oral squamous cell carcinoma (A) Gross image showing ulcerated, erythematous areas, with white patches and irregular margins on the lateral border of the tongue. (B) Histopathology image showing well-differentiated squamous cell carcinoma having nests of squamous islands with central keratinization (H&E, 40x).

Among these 53 patients with oral squamous cell carcinoma, a history of tobacco chewing was reported in 30 individuals (56.6%), making it the most commonly observed habit. Smoking was documented in 15 cases (28.3%), while betel nut chewing was noted in eight cases (15.1%). These findings indicate that tobacco chewing was the predominant risk habit in the study population (Figure 1).

In our study, among the geriatric gastrointestinal tract cancer cases, adenocarcinomas (all subtypes combined) account for 10 out of 17 cases (58.8%), making them the predominant histological category. Other subtypes were observed less frequently (Table 4).

**Table 4:** Differentiation of geriatric cancer in the gastrointestinal tract

Sr. No.	Histopathological diagnosis	Cases	Percentage (%)
1	Adenocarcinoma moderately differentiated	06	35.4
2	Metastatic carcinoma	06	35.4
3	Adenocarcinoma well-differentiated	02	11.8
4	Neuroendocrine carcinoma	01	5.8
5	Diffuse-type adenocarcinoma	01	5.8
6	Invasive mucinous adenocarcinoma	01	5.8
Total		17	100

**Figure 2:**
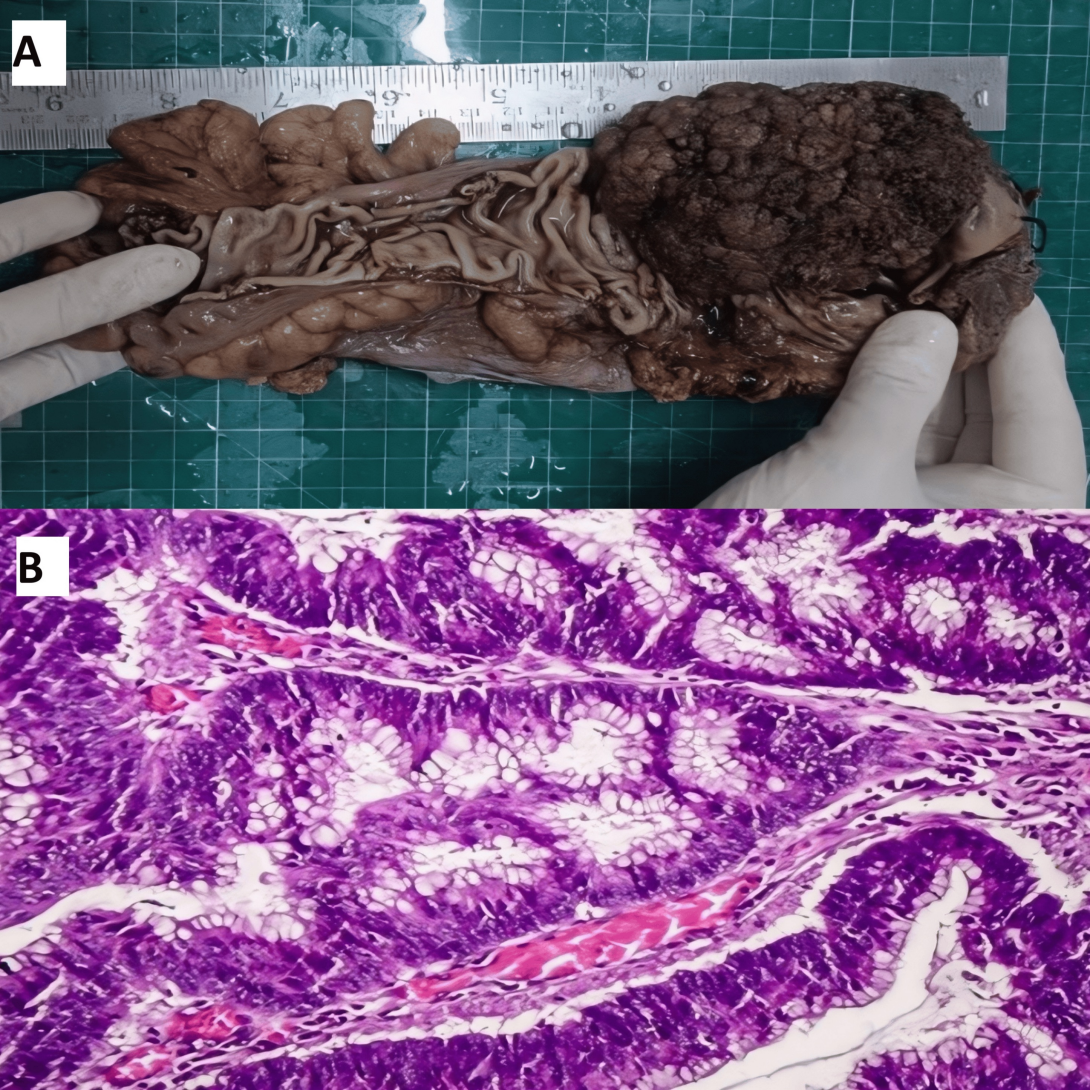
Colonic adenocarcinoma (A) Gross image showing a colonic mass presenting as an ulcero-proliferative growth. (B) Histopathology image showing intestinal adenocarcinoma showing large invasive glands lined by dysplastic columnar mucinous cells with hyperchromatic nuclei and mitosis (H&E, 40x).

Figure 2 presents the colonic adenocarcinoma.

Among the 13 breast lesions analyzed, the most prevalent malignant condition was invasive ductal carcinoma, accounting for 61.5% of cases, followed by invasive lobular carcinoma, which represented 23% of cases (Table 5).

**Table 5:** Distribution of breast lesions according to histopathological diagnosis

Sr. No	Histopathological diagnosis	Number of cases	Percentage (%)
1	Invasive ductal carcinoma of the breast	08	61.5
2	Invasive lobular carcinoma of the breast	03	23
3	Invasive breast carcinoma	02	15.5
Total		13	100

**Figure 3:**
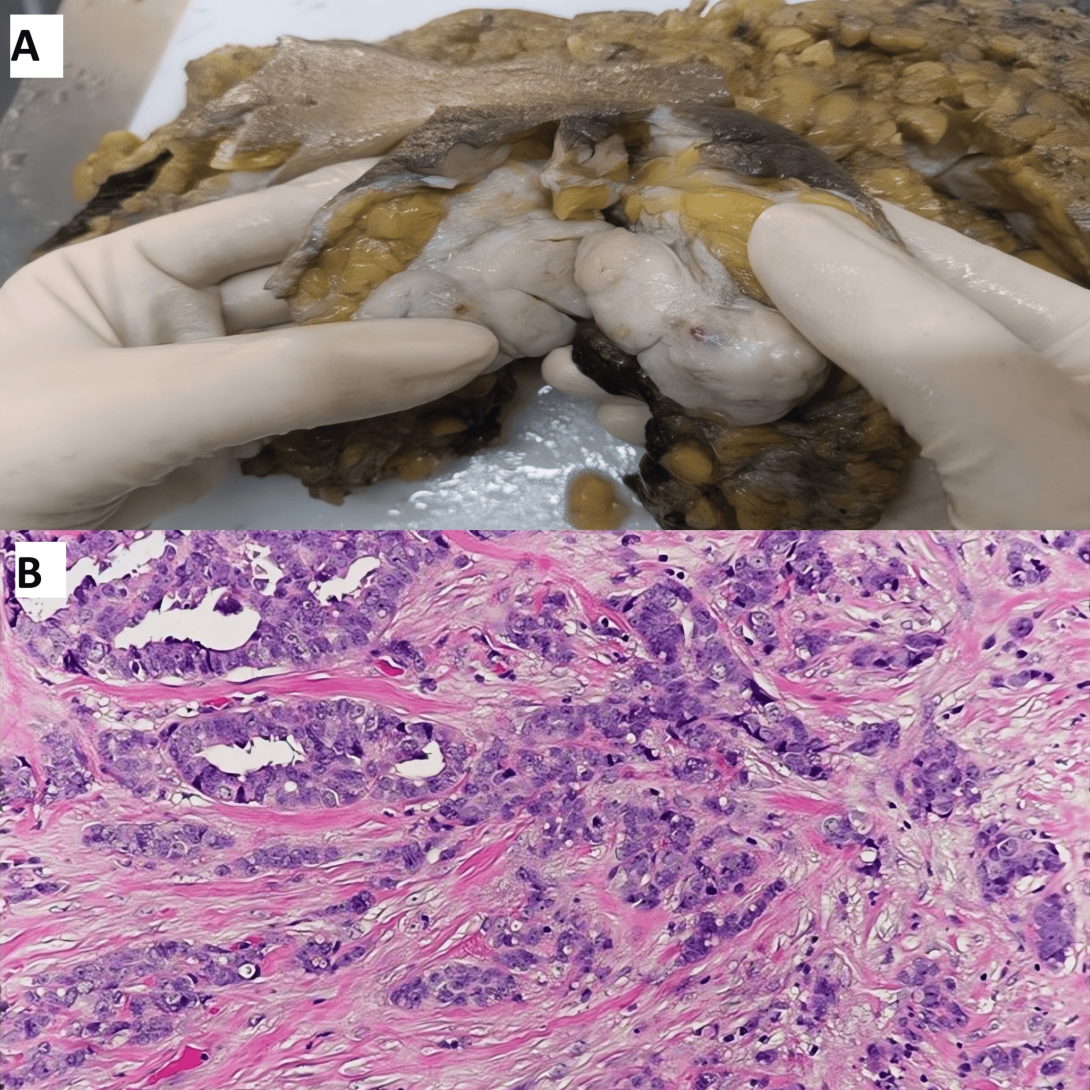
Breast ductal carcinoma (A) Gross image showing a mass lesion in the breast having a white firm cut surface. (B) Histopathology image showing invasive ductal carcinoma, breast, having infiltrative tubules, small nests and a trabecular pattern surrounded by desmoplastic stroma (H&E, 40x).

Figure 3 presents breast ductal carcinoma.

## Discussion

This study includes 111 patients who had been diagnosed with cancer and were aged 60 and above and subsequently evaluated after a biopsy for histopathological diagnosis. The patient profile was studied in detail to evaluate the incidence in relation to age, sex, and lesion location. A detailed study of histological features and tumor grading was carried out.

Among the 53 oral squamous cell carcinoma cases, histopathological evaluation revealed considerable variation in tumor differentiation. Moderately differentiated tumors were the most common, observed in 25 cases (47.2%), reflecting their intermediate biological behavior. Well-differentiated tumors accounted for 20 cases (37.7%), representing a significant portion of lesions with relatively favorable features. Poorly differentiated tumors were less frequent, seen in eight cases (15.1%), and are associated with a higher potential for aggressive progression. This distribution highlights the histological heterogeneity of oral cancers in the geriatric population and underscores the importance of thorough tumor grading for prognostic assessment and treatment planning.

In the present study, the majority of oral cavity cancer cases were in the 61-70-year age group, representing 66.4% of patients, followed by 26.0% in the 71-80-year age group and 7.5% in those aged 81-90 years. Oral lesions demonstrated a marked male predominance, with 77% of patients being male and 23% female, corresponding to a male-to-female ratio of 3.6:1. In comparison, other studies have reported varying proportions in the 61-70 year age group, with Rahul et al. (10.06%), Modi et al. (16.60%), and Patro et al. (12%), and is likely influenced by higher exposure among males to risk factors, such as tobacco use, including both smoking and chewing forms [15-17] (Table 6).

**Table 6:** Comparative analysis of age incidence of all oral cavity lesions Source: [15-17]

Age group (in years)	Rahul et al., No. (%)	Modi et al., No. (%)	Patro et al., No. (%)	Present study, No. (%)
61-70	50 (10.06)	43 (16.60)	10 (12.0)	35 (66.46)
71-80	12 (2.41)	21 (8.11)	02 (3.0)	14 (26.04)
81-90	12 (2.41)	-	-	04 (7.5)

It was observed that squamous cell carcinoma was the commonest variety of malignancy in all the studies. Thus, its incidence is comparable in all the studies, including the present study. All 53 oral lesions in the present study were diagnosed as squamous cell carcinoma (100%). In India, oral squamous cell carcinoma occurs predominantly in males, with a reported male-to-female ratio of approximately 2.9:1. The buccal mucosa is the most commonly affected site, followed by the tongue. This high incidence is largely attributed to lifestyle and environmental risk factors, including tobacco use (both smoking and smokeless forms), betel-quid chewing, alcohol consumption, and poor oral hygiene. Consistent with these patterns, the present study found squamous cell carcinoma to be the most prevalent malignant condition among the geriatric population evaluated [16,18,19] (Table 7). 

**Table 7:** Comparison of histopathological varieties of oral malignant tumors Source: [16,18,19]

Histopathological diagnosis	Modi et al. [16], 2021-2023 (Rajasthan, India)	Gupta et al. [18], 2020-2021 (Jammu and Kashmir, India)	Bastakoti et al. [19], 2018-2019 (Nepal)	Present study, 2023-2025 (Rajkot, India)
Squamous cell carcinoma	92.74%	71.42%	95.50%	100%

Although breast and gastrointestinal malignancies were identified in this cohort, the sample sizes for these sites were small (<20 cases each). Invasive ductal carcinoma was the most frequent breast malignancy (61.5%), while adenocarcinoma predominated among gastrointestinal tumors (58.8%). Due to these limited numbers, no meaningful statistical or comparative analysis could be conducted, and detailed tables comparing these findings with other studies were intentionally omitted. This approach allows the manuscript to maintain clarity and focus on oral cavity cancers, which represent the largest and most clinically significant portion of the cohort.

The less frequently encountered tumors comprised lobular carcinoma of the breast, metastatic colon carcinoma, basal cell carcinoma, small cell carcinoma of the lung, papillary thyroid carcinoma, Hodgkin's lymphoma, and other rare malignancies, each accounting for 2.7% or fewer of cases.

This study is limited by its single-center design and relatively small sample size, which may affect generalizability. Furthermore, long-term outcomes and survival data were not evaluated. Future multi-center studies with larger cohorts and follow-up data are needed to validate these findings and inform national geriatric cancer management strategies.

## Conclusions

The present study shows that oral cavity carcinoma, particularly squamous cell carcinoma, is the most frequently encountered malignancy among the geriatric patients evaluated. Most cases were observed in the 61-70-year age group, with a clear male predominance, which is consistent with findings reported in previous studies. These observations reflect the continuing burden of oral cancer in elderly individuals, particularly among those with exposure to established risk factors such as tobacco use. Although the study included a limited number of cases, it highlights the importance of histopathological evaluation in confirming the diagnosis and supporting appropriate clinical management of oral cavity malignancies in the geriatric population. These findings highlight the importance of early detection, targeted screening, and tailored treatment approaches for geriatric cancers in India, and reinforce the role of histopathological analysis in guiding clinical decision-making.
